# Asiaticoside attenuates hyperoxia-induced lung injury *in vitro* and *in vivo*

**DOI:** 10.22038/ijbms.2019.35913.8556

**Published:** 2019-07

**Authors:** Jia-wen Dang, Xiao-ping Lei, Qing-ping Li, Wen-bin Dong

**Affiliations:** 1 Department of Newborn Medicine, The Affiliated Hospital of Southwest Medical University, Luzhou, Sichuan 646000, China

**Keywords:** Apoptosis, Asiaticoside, Hyperoxia, Inflammation, Lung injury, Premature

## Abstract

**Objective(s):**

Asiaticoside (AS) displays anti-inflammation, and anti-apoptosis effect, but the role of AS in hyperoxia-induced lung injury (HILI) treatment is undefined. Therefore, the aim of this study was to investigate the effects of AS on HILI on premature rats and alveolar type II (AEC II) cells.

**Materials and Methods:**

Sprague-Dawley premature rats (n=25/group) were exposed to 80% O_2_ with or without AS. Then, we detected 80% O_2_-induced lung injury and survival rate of premature rat. We tested the concentration of malondialdehyde (MDA), myeloperoxidase (MPO), total antioxidant capacity (TAOC), tumor necrosis factor α (TNF-α), interleukin 6 (IL-6), and interleukin 1β (IL-1β) in premature rats’ blood. Then, the AEC II cell apoptosis was observed by Hoechst 33258 staining and flow cytometry. Simultaneously, nuclear factor (erythroid-derived 2)-like 2 (Nrf2) signaling pathway was measured by Western blot.

**Results:**

Our results found that AS-treated group rats had significantly higher survival rates than 80% O_2_ group at day 14 (*P<*0.05). AS protected HILI, decreased the MPO and MDA concentration, and reversed TAOC level (*P<*0.05). AS also downregulated the levels of TNF-α, IL-1β, and IL-6 in the premature rat’s blood (*P<*0.01). Moreover, AS markedly attenuated AEC II cell apoptosis and increased Nrf2 and Heme oxygenase 1 (HO-1) expression in the nucleus (*P<*0.05).

**Conclusion:**

AS showed protective effects on premature rats of HILI *in vitro* and *in vivo*. AS can potentially be developed as a novel agent for the treatment of HILI diseases.

## Introduction

Bronchopulmonary dysplasia (BPD) is a common disease in premature baby and there is no effective agent for BPD therapy. The pathological change induced by BPD is hyperoxia induced lung injury (HILI) in immature lung tissue, which is caused by mechanical ventilation and long-term hyperoxia treatment. However, the pathogenesis of BPD is extremely complex and the exact mechanism remains unclear (1). Therefore, the underlying mechanisms need to be elucidated. An agent that has the potential to treat hyperoxia-induced oxidative injury of the immature lung is needed.

Oxygen therapy is a strategy for critically ill patients; hyperoxia can cause acute lung injury, thereby contributing to BPD (2). Substantial evidence from animals and humans show that inflammation response (3) and oxidative stress injury (4) play a very important role in BPD development. Disruption of inflammation and anti-inflammation balance is an important mechanism contributing to HILI, and inhibiting the NF-kappaB activity attenuates HILI in adult mice (5). In addition, hyperoxia exposure induces oxidative stress, which may activate necroptosis, and this is involved in the pathology of HILI; thus, strategies targeting necroptosis may become promising treatments for HILI (6). Therefore, target for inflammation response and oxidative stress can be a feasible treatment for HILI.

Several compounds isolated from natural products exhibit significantly protective activity on lung injury via anti-inflammatory, antioxidative, and anti-apoptosis effects (7-9). Asiaticoside (AS), a naturally triterpenoid saponin extracted from *Centella asiatica*, has anti-apoptotic and anti-inflammatory activities (10,11). Aβ_1-42_-induced apoptosis cells were markedly decreased by AS via elevating the ratio of Bcl-2/Bax (12). AS can also attenuate cell apoptosis induced by Aβ_1-42_ through inhibiting the TLR4/NF-κB signaling pathway (13). Moreover, recent studies reported that AS prevented hypoxia-induced pulmonary hypertension (hypoxic PH) by blocking transforming growth factorbeta1/SMAD family member 2/3 signaling pathway (11). Therefore, we hypothesized that AS has a significant protective effect on HILI.

Considering the potential significant protective effects of AS on lung tissue, the aim of this study was to investigate the protective effects of AS on HILI. Rat and cell models of HILI were used to determine the potential protective effects of AS and the associated mechanisms. Our results may provide a reasonable theoretical foundation for the development of HILI treatment and may present evidence of the potential of AS as a novel agent for the treatment of BPD in premature babies. 

## Materials and Methods

***Regents***

Asiaticoside (AS, Figure 2A,), was purchased from MedChemExpress (Cat No HY-N0439, Monmouth Junction, NJ, USA) with a purity >98% (HPLC) and dissolved in normal saline (NS). TUNEL apoptosis assay kit and Hoechst 33258 apoptosis assay kit were purchased from Beyotime (Haimen, Jiangsu, China). Annexin V-FITC apoptosis detection kit was purchased from Dojindo Chemical Technology Co, Ltd. (Shanghai, China). 

***Experimental animals***

**Figure 1:**
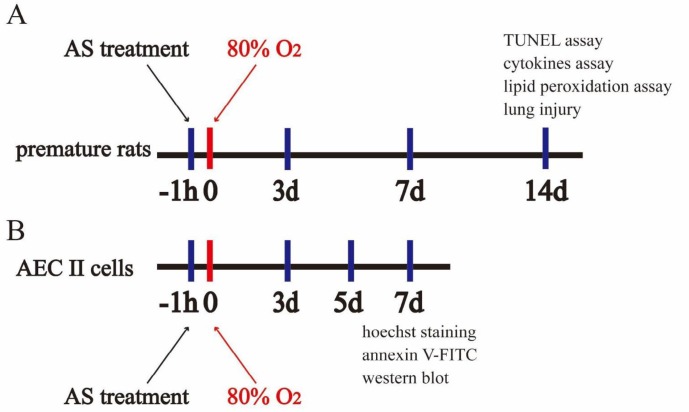
Schematic diagram of the experimental procedure

**Figure 2:**
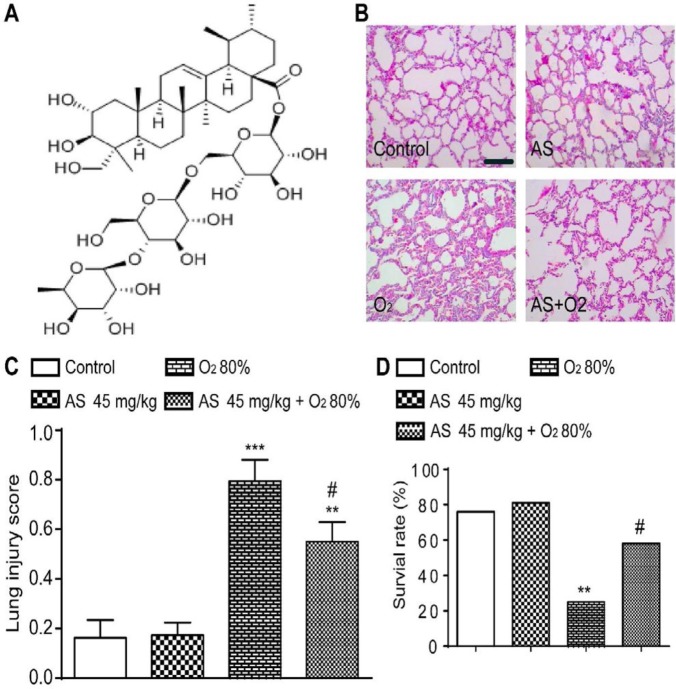
Asiaticoside (AS) alleviated hyperoxia-induced lung injury and improved the survival rate

Eight-weeks-old specific pathogen free (SPF) Sprague-Dawley rats were purchased from the Experimental Animal Center of Southwest Medical University (Luzhou, Sichuan, China, Certificate No SCXK2013-24). The same number of male and female rats were housed in the cage for mating. Then, we checked the vaginal plugs of female rats; this was designated as the first day of female rats’ pregnancy. The premature rats were obtained from female rats day 21 of gestation by caesarean section (full term is 22–23 days)(14), and the spontaneous-born rats were excluded. These surviving premature rats were used for experiments, and the procedure showed in Figure 1A. All premature rats were randomly divided into four groups (n=25 in each group) and subjected to treatment for 3, 7, and 14 days, as follows. Control group comprised the premature rats treated with NS (1 ml/kg); AS group comprised the premature rats treated with AS (45 mg/kg, Figure 2A), previous study found that 45 mg/kg could evidently reduce lung injury *in vivo* (15). Model group comprised the premature rats raised with O_2_ (80%) as previously described with modification (16, 17), HILI was induced by keeping newborn rats in a chamber with continuous exposure to 80% O_2_ and the oxygen level inside the chamber was monitored continuously with a Ceramatec (MAXO2) oxygen analyzer. The therapy group comprised the premature rats treated with AS (45 mg/kg)+O_2_ (80%). The NS and AS were administrated intraperitoneally at 1 hr before 80% O_2_ treatment. All animal experiments were performed strictly according to the Ethics Committee of the Affiliated Hospital of Southwest Medical University (20170921001), and in accordance with “Principles of Laboratory Animal Care” (NIH publication No 85-23, revised 1985, http://grants1.nih.gov/grants/olaw/references/phspol.htm).

***Experimental cells***

For alveolar type II (AEC II) cells culture, lung tissues were rapidly removed under sterile conditions, and type II cells were isolated using the method, as previously described, with modifications (18). The lung tissues of premature rats were cut into pieces (1 mm^3^), digested by 0.25% trypsin, and centrifuged at 1000 × g for 4 min to remove fibroblasts, The AEC II cells were cultured in DMEM supplemented with 10% FBS (Gibco), 1% penicillin, and streptomycin (Beyotime) in 5% CO_2_ at 37 ^°^C in a humidified incubator. The culture was supplemented with fresh medium every 2 to 3 days. The cells were divided into four groups, which were cultured under different conditions, such as control group treated with cell medium, AS group treated with AS (50 μM), model group treated with O_2_ (80%), and therapy group treated with AS (50 μM)+O_2_ (80%). All cells in each group were harvested for the subsequent experiments, and the procedure showed in Figure 1B. 

***Histologic examination of lung tissues***

Under deep anesthesia with pentobarbital sodium (60 mg/kg, IP), the right lung tissues were fixed in 4% paraformaldehyde and embedded in paraffin. Lung sections (5 μm) were stained with hematoxylin and eosin (HE) and examined under a light microscope. Lung injury was evaluated and scored by an investigator who was blinded to experimental grouping, as previously described (19), and the lung damage was assessed using a two-point scale, ranging from 0 to 1.

***Measurements of malondialdehyde (MDA), myeloperoxidase (MPO), and total antioxidant capacity (TAOC)***

Under deep anesthesia (pentobarbital sodium, 60 mg/kg, IP), the right lung was excised and immediately 100 mg of the lung was homogenized and fluidized in extraction buffer to obtain 5% of homogenate. The effects of AS and/or hyperoxia on the levels of MDA, TAOC, and MPO were determined in the lung tissues by using the MDA and MPO assay kit purchased from Thermo Fisher Scientific Inc. (MA5-27559 and MA1-20073, Waltham, MA, USA). TAOC kit was obtained from Jiancheng Biotechnology (A015-2, Jiancheng, Nanjing, China).

***Inflammatory cytokines in the blood of premature rats***

The levels of TNF-α, IL-6, and IL-1β in the rats’ blood harvested before lung tissues collection were detected using TNF-α, IL-1β, and IL-6 ELISA kits obtained from R&D (RTA00, RLB00, and R6000B, R&D systems, Minneapolis, MN, USA). All the experiments were performed followed manufacturer’s instruction. 

***TUNEL assay of lung tissues of premature rats***

The TUNEL assay was performed using the *In Situ* Cell Death Detection Kit (11684817910, Roche Diagnostics, Indianapolis, IN, USA). The genomic DNA between nucleosomes was cut off by DNA endonucleases that were activated when cells apoptosis occurred due to stimuli (20). In sync with genomic DNA breakage, the exposed 3’-OH were combined with dUTP labeled with Biotin under the catalysis of terminal deoxynucleotidyl transferase and then combined with Streptavidin labeled with horseradish peroxidase (HRP) (21). Finally, apoptotic cells were displayed by DAB chromogenic under the catalysis of HRP and observed by light microscopy. The TUNEL assay was performed in accordance with agent instruction and apoptotic cells were observed via inverted microscopy at ×400 magnifications (CKX41, Olympus Corporation, Shinjuku-ku, Tokyo, Japan).

***Apoptosis assay of AEC II cells by hoechst 33258 staining***

Morphological analysis of apoptotic cells was performed by Hoechst 33258 staining (C0003, Beyotime, Nanjing, China) in accordance with a previously described method (22). First, the AEC II cells were cultured in 6-well plates with 5% CO_2_ at 37 ^°^C and treated under different conditions and/or with different agents. The cells in control group were cultured in cell medium. The cells in AS group were treated with only AS (50 μM). The cells in model group were cultured with only O_2_ (80%). The cells in therapy group were processed by AS (50 μM)+O_2_ (80%) for 3, 5, and 7 days. Next, the medium was removed and washed with pre-cooling PBS for 15 min before incubation with fixative solution at 500 μl/well for 10 min. The cells were repeatedly washed with PBS for 15 min and subsequently treated with Hoechst 33258 staining solution with 500 μl/well for 5 min in a dark room. Finally, the staining solution was removed from plates and repeatedly washed by PBS for 15 min. The cells were observed by fluorescence microscopy at×400 magnifications (AMG EVOS, Thermo Fisher Scientific Inc., Waltham, MA, USA), and the effects of AS were evaluated by the ratio of apoptotic cells. 

***Detection of apoptotic cells by flow cytometry***

The cellular apoptosis of AEC II cells was detected by flow cytometry with Annexin V-FITC apoptotic detection kit (C1062L, Beyotime, Nanjing, China) in accordance with the manufacture’s instruction. Firstly, the cells were seeded in six-well plates at a density of 1×10^5^ and treated under different conditions and/or agents. The cells in the control group were cultured with cell medium, the cells in AS group were treated with only AS (50 μM), the cells in model group were cultured with only O_2_ (%), and the cells in therapy group were processed by AS (50 μM)+O_2 _(%) for 3, 5, and 7 days, respectively. The cells were washed with pre-cooling PBS for 10 min and digested by 0.25% trypsin for 3 min to create cell suspensions with medium, which were subsequently centrifuged at 1,500 × g for 3 min at room temperature. Furthermore, the cells were resuspended in Annexin V-FITC binding buffer. Annexin V-FITC at 5 μL and/or PI at 5 μl was carefully added to the cells, which were then incubated for 15 min in the dark room. The quantity of apoptotic cells were detected by Flow Cytometr (FACSCalibur, BD Biosciences, San Diego, CA, USA).

***Western blot***

The expressions of associated protein in Nrf2 signaling pathway were measured by Western blot. AEC II cells were seeded at a density of 2.0×10^5^ cells/well on 6-well plates, and the cells in control group were cultured with cell medium. The cells in AS group were treated with only AS (50 μM), the cells in model group were cultured with only O_2_ (80%), and the cells in therapy group were processed by AS (50 μM)+O_2_ (80%) for 3, 5 and 7, days, respectively. Then, the cells were lysed in RIPA lysis buffer, and the lysates were centrifuged at 10000 × g for 20 min at 4 ^°^C. All protein samples (25 μg) were electrophoresed on 10% SDS-PAGE gel for separation and electrotransfer to PVDF membranes. The membrane was then blocked with 5% non-fat milk in Tween-PBS buffer for 1 hr, and then, the membranes were incubated with primary antibody: anti-Nrf2 ((D1Z9C) XP^®^ Rabbit mAb,), anti-Lamin B1 (Lamin B1 (D4Q4Z) Rabbit mAb, 1:1000), anti-HO-1 (HO-1 (D60G11) Rabbit mAb, 1:1000), and β-actin (β-actin (13E5) Rabbit mAb, 1:1000) at 4 ^°^C overnight before incubation with secondary antibody (Beyotime, Haimen, Jiangsu, China, 1:5000) for 1 hr at room temperature. The specific protein band was then detected on ECL films using the ChemiDoc image analyzer (Bio-Rad, Hercules, CA, USA) to collect images, and measurement was conducted by using Image J 3.0 software.

***Statistical analysis***

All data were represented as mean±standard deviation (SD). The normal distribution of behavioral data was assessed using the Shapiro-Wilk’s test. Statistical analyses were carried out using the SPSS 20.0 statistical software (SPSS Inc, Chicago, IL, United States). The difference among groups were analyzed by one-way ANOVA followed by *post hoc* Bonferroni test. Chi-square test was applied for survival rates, and *P*<0.05 was considered to be statistically significant.

## Results

***AS attenuated 80% O***_2_ ***induced lung injury and improved survival rate***

On histological examination, lungs of animals exposed to hyperoxia (80% O_2_) showed simplified alveoli characterized by larger, fewer, and less complex alveoli as compared with the animals in the control group (*P*<0.01 and *P*<0.001), whereas AS (45 mg/kg) protected lung tissues injury from 80 % O_2 _(*P*<0.05) (Figures 2B-C). The survival rates of the control and AS groups were (19/25) and (20/25), respectively. Simultaneously, AS pretreated group rats had significantly higher survival rates (14/25) than 80% O_2_ group (6/25) at day 14 (*P*<0.01 and *P*<0.05) (Figure 2D).

***AS reduced the levels of MDA and MPO, and elevated the level of TAOC in the blood of premature rats***

We then investigated the effects of AS on the levels of MDA, MPO and TAOC in premature rats blood. As shown in Figures​ 3A-C, 80% O_2_ significant increased levels of MDA and MPO but decreased TAOC level in the premature rats compared with the control rats (*P*<0.05 and *P*<0.01). However, treatment with AS (45 mg/kg) markedly down-regulated the levels of MDA and MPO but elevated TAOC level compared with the 80% O_2_ treated rats (*P*<0.05). 

***AS decreases the levels of TNF-α, IL-1β and IL-6 in premature rats***

To observe the anti-inflammation effect of AS, we tested the proinflammatory factors. We found that 80% O_2_ increased the levels of TNF-α, IL-1β, and IL-6 compared with that of control group (*P*<0.01), whereas AS (45 mg/kg) significantly down-regulated the levels of TNF-α, IL-1β, and IL-6 in the blood of premature rats at 3, 7, and 14 days induction by 80% O_2_(*P*<0.05 and *P*<0.01 ) (Figure ​4). 

***AS attenuates the lung cells’ apoptosis in premature rats***

To further observed the protective effect of AS, apoptosis of lung cells treatment with O_2_ (80%) or AS (45 mg/kg) in the premature rats were detected by TUNEL assay. The apoptotic cells (arrow indication) were significantly increased by 80% O_2_ at 3, 7, and 14 days in premature rats compared with control rats (Figure 5). However, the apoptotic cells in lung tissues in premature rats were decreased by AS (45 mg/kg) treatment at 3, 7, and 14 days. 

***AS attenuates cells apoptosis induced by hyperoxia in AEC II cells***

To further examine the effect of AS on the AEC II cells *in vitro,* we performed Hoechst 33258 staining*.* The results from Hoechst 33258 staining showed that the apoptotic cell’s ratios were markedly elevated by 80% O_2_ treatment at 3, 5 and 7 days compared with control cells (*P*<0.01) (Figure 6). Nevertheless, AS pretreatment significantly decreased apoptotic cells induced by O_2_ (80%) at 3, 5, and 7 days. These results have proved that AS can protect AEC II cells from apoptosis induced by hyperoxia *in vitro *(*P*<0.01)*.*

In order to quantify the number of apoptotic cells, flow cytometry was applied to determine cell ratio in the early or late stages of apoptosis (Figure 7). The apoptotic ratio was significantly increased by O_2_ (80%) treatment at 3, 5 and 7 days compared with control cells (*P*<0.01), whereas AS (50 μM) markedly decreased the ratio of apoptotic cells at 3, 5, and 7 days compared with O_2_ (80%) group (*P*<0.01). 

***AS increases Nrf2 and HO-1 expressions in AEC II cells***

The effect of AS on Nrf2 signaling pathway was investigated by Western blot analysis. As shown in Figure 8, 80% O_2_ decreased Nrf2 and HO-1 expressions at days 3, 5, and 7 compared with control group(*P*<0.05 and *P*<0.01). However, AS (50 μM) markedly elevated the levels of Nrf2 and HO-1 in cells treated with O_2_ (80 %) at days 3, 5 and 7 compared with the 80% O_2_ group (*P*<0.05 and *P*<0.01). 

## Discussion

In the present study, AS can markedly attenuate HILI, improve the premature rats survival rate, and decrease the levels of cytokines TNF-α, IL-1β, IL-6, MDA, MPO, and reverse TAOC level in the blood of premature rats after treatment with hyperoxia. AS also attenuated apoptosis in AEC II cells. Furthermore, AS up-regulated the protein expressions of Nrf2 and HO-1 in AEC II cells. These results indicated that AS has a protective effect on HILI in rats through antiinflammation and antiapoptosis pathways by up-regulating Nrf2 and HO-1 expression.

BPD is a common neonatal complication, and it especially occurs in premature infants with gestational ages of less than 26 weeks and weigh less than 1000 g (23). The pathology of BPD mainly includes fibrosis and local pulmonary enlargement (24). This can damage lung and respiratory tract in premature babies, and it lacks effective treatment (25). The potential mechanism for BPD is HILI, but the pathogenesis has not been fully clarified. Previous studies have proven that the cascade response of inflammatory factors induced by oxygen toxicity, pulmonary edema, sepsis, and maternal infection caused original immature lung damage (26-28). 

Proinflammatory factors play a critical role in many pathological diseases. TNF-α is a cell signaling cytokine involved in systemic inflammation, and it induces the acute phase reaction. TNF-α contributes to fever, cell apoptosis, and inflammation (29). Therefore, dysregulation of TNF-α production promotes the development of many human diseases, such as Alzheimer’s disease, cancer, and inflammatory lung disease (30, 31). IL-6 possesses both proinflammation and anti-inflammation effects. IL-1β is also a critical mediator in inflammatory response. In our present study, 80% O_2_ increased these inflammation cytokines consistent with a previous study, whereas pretreatment with AS can decrease the proinflammatory cytokine production.

**Figure 3:**
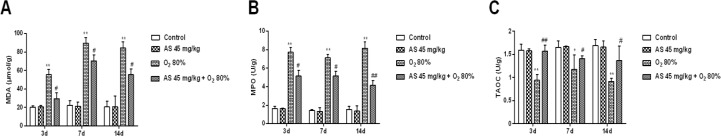
Asiaticoside (AS) attenuated hyperoxia-induced lipid peroxidation injury in lung tissues

**Figure 4:**
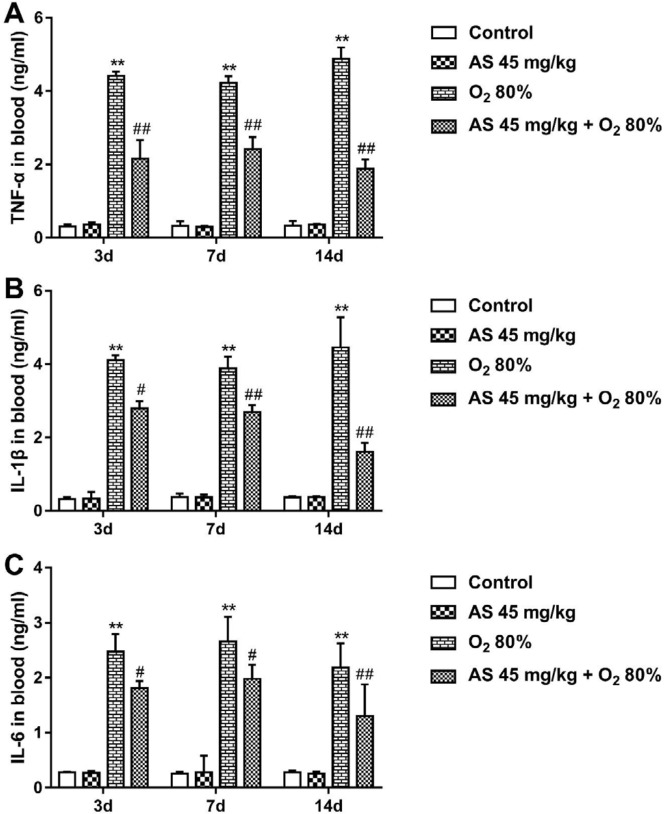
Asiaticoside (AS) attenuated hyperoxia-induced inflammation TNF-α, IL-1β, and IL-6 induced by O_2_ in blood of premature rats detected by ELISA. (A) 45 mg/kg AS decreased 80% O_2_ induced TNF-α level on days 3, 7 and 14. (B) 45 mg/kg AS decreased O_2_ (80 %) induced IL-1β level in lung tissues on days 3, 7 and 14. (C) 45 mg/kg AS decreased IL-6 level after O_2_ (80 %) exposure in lung tissue on day 3, 7 and 14. The results were represented as mean±SD, n=4 in each group. **P<0.01 vs control group, #*P<*0.05 and ##*P<*0.01 vs 80 % O_2_-treated group

MDA is a natural product of lipid oxidation. Lipid oxidation occurs when organisms undergo oxidative stress injury. Lipid peroxidation of polyunsaturated fatty acids produce a series of complex compounds, including MDA; the level of lipid oxidation can be evaluated by detecting the level of MDA (32). Therefore, the determination of MDA is widely used as an indicator in evaluating oxidative stress. In this study, 80% O_2_ increased MDA level, and AS decreased MDA level. This finding indicated that 80% O_2_ induced oxidative stress injury, and AS protected lung tissues and cells from oxidative stress injury by decreasing MDA. MPO is a functional marker of neutrophils. MPO activity change indicates the function and activity state of neutrophil polymorphonuclear leukocytes (33). Under pathological conditions, MPO catalyzes the formation of excessive oxidants, thereby resulting in oxidative stress damage. MPO is also involved in many processes that regulate the inflammatory response (34). The TAOC in the serum is mainly the total antioxidants in the non-enzymatic system in the serum and the few small molecular weight antioxidants in the enzymatic system. It is one of the important indexes that reflect the antioxidation of the body (16). Therefore, we detected the expression of MPO, and we found that 80% O_2_ up-regulated the MPO level. AS treatment down-regulated MPO level and MDA level, However, TAOC level was significantly increased by AS. The lung tissues was severely damaged in premature rats, and the findings were in accordance with the previous reports (35). However, AS effectively inhibited lung damage and decreased the blood levels of TNF-α, IL-6, IL-1β, MDA, and MPO induced by hyperoxia in premature rats (Figures 3 and 4). These studies have proven that AS may prevent lung injury by down-regulating inflammation cytokines production in premature rats. 

**Figure 5:**
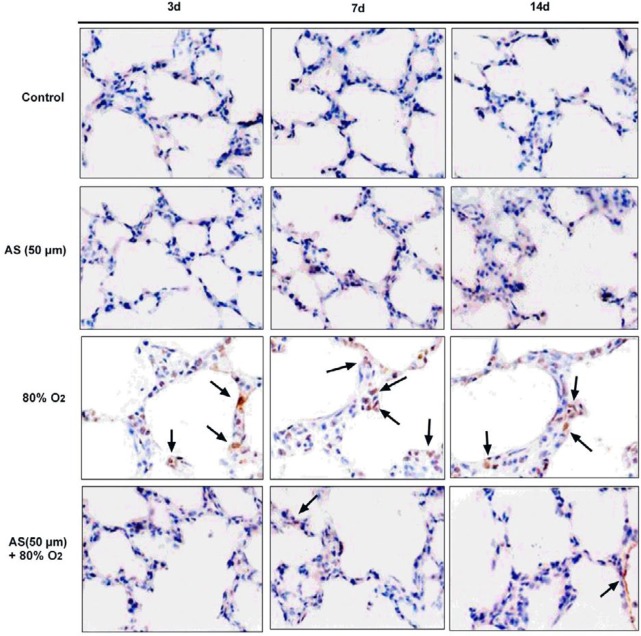
Asiaticoside (AS) mitigated 80% O_2_-induced cells apoptosis in lung tissues in premature rats

Cell apoptosis in tissues is also an indicator of lung injury (36). TUNEL assay results showed that apoptotic cells were markedly increased at 3, 5, and 7 days, but AS effectively reduced the elevated apoptotic cells in premature rats mediated by hyperoxia (Figure 5), thereby suggesting that AS could obviously protect lung cells from apoptosis in premature rats. In addition, the protection of AS on hyperoxia-induced AEC II cells injury *in vitro* was further investigated by flow cytometry. These results showed that AS can significantly decrease the ratio of apoptotic cells in the early and late stages of apoptosis, thereby suggesting that AS also protects AEC II cells* in vitro*. Primary alveolar epithelial type II cells from mouse (37) and rat (38) have been reported to trans differentiate to alveolar epithelial type I-like cells before 7 days with a great phenotypical changes of the cells. Therefore, the isolated primary AEC II cells at day 3, 5, and 7 were used for long-term toxicological experiments. Simultaneously, the molecular mechanism of AS in protecting lung tissue was investigated by Western blot analysis in AEC II cells. The nuclear factor Nrf2 signaling pathway is a critical transcription factor that protects against exogenous stimuli and toxic antioxidant responses (39). The regulation of Nrf2 signaling pathway is likely to be closely related to a variety of physiological functions, pathological changes, diseases, and cancer (40, 41). Nrf2 signaling pathway plays a critical role in inflammation, oxidative stress, and lung disease (42-44). Our results showed that 80% O_2_-induced lung injury down-regulated the levels of Nrf2 and HO-1. However, AS markedly increased the expression of Nrf2 and HO-1 compared with 80% O_2_ group, thereby indicating that AS could enhance Nrf2 signaling pathway to protect AEC II cells *in vitro. *

There are some limitations in our study. First, the effect of AS and a reference drug for treatment of hyperoxia-induced lung injury should be compared, such as dexamethasone. Second, we did not observe the markers for AEC II and AEC-I cells in premature rats. Therefore, further investigations are required to determine the complete mechanisms underlying these phenomena. However, we still have reason to speculate that the improvement of AS on HILI in premature rats. 

(A) Schematic diagram of the study in premature rats. AS pretreatment 1 hr before 80%O_2_ exposure, and lipid peroxidation, cytokines and TUNEL assay were performed at day 3, 7, and 14. (B) Schematic diagram of the AEC II cells. AS pretreatment 1 hr before 80% O_2_ exposure, ACE II cell apoptosis and Nrf2 signaling pathway evaluated at day 3, 5, and 7

AS: Asiaticoside

(A) Chemical structure of AS. (B) Representative histology images of lung tissues at day 14 post 80% O_2_ and AS exposure. Scar bar=200 μm. (C) Lung injury scores of four groups, **P<0.01, ***P<0.001 vs the control group, #*P<*0.05 compared with the 80% O_2_ group, the data are represented as mean±SD, n=6 in each group. (D) Simultaneously, AS treated group rats had significantly higher survival rates than 80% O_2_ group at day 14, **P<0.01 vs the control group, #*P<*0.05 compared with the 80% O_2_ group, n=25 in each group

MDA, MPO, and TAOC levels in lung tissues were measured after 80% O_2_ and AS treatments. (A) 45 mg/kg AS decreased 80 % O_2_-induced lung tissue MDA level on days 3, 7, and 14. (B) 45 mg/kg AS decreased 80% O_2_-induced lung tissue MPO expression on day 3, 7 and 14. (C) 45 mg/kg AS increased TAOC level after O_2_ (80 %) exposure in lung tissue on day 3, 7 and 14. The data are represented as mean±SD, n=4 in each group. *P<0.05 and **P<0.01 vs control group, #*P<*0.05 and ##*P<*0.01 vs 80% O_2_-treated group

MDA: malondialdehyde; MPO: myeloperoxidase; TAOC: total antioxidant capacity

The 80% O_2_ increased apoptosis cells in lung tissues, whereas treatment with 45 mg/kg AS protect lung cells from apoptosis. The black arrows indicate apoptotic cells. Scar bar=100 μm

**Figure 6:**
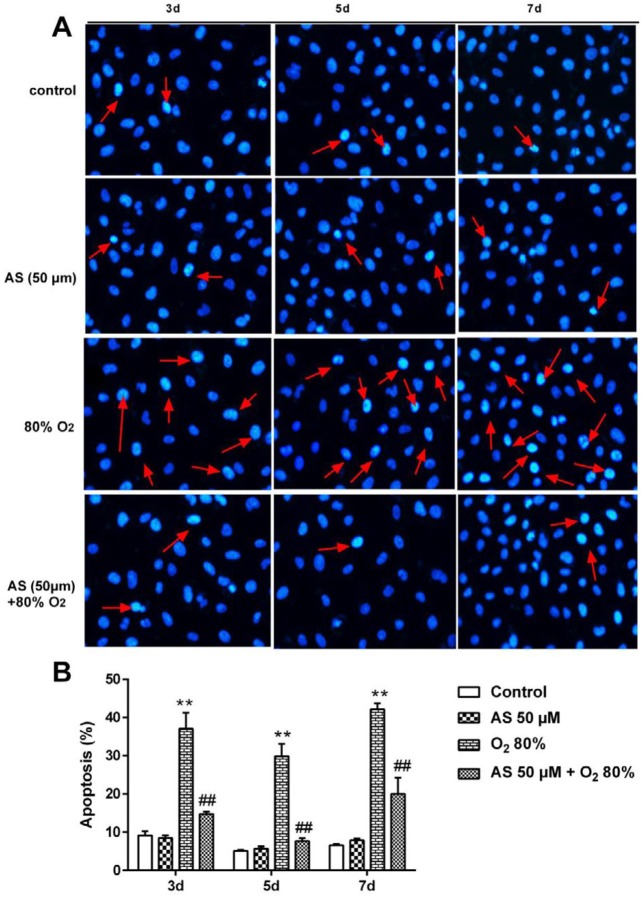
Asiaticoside (AS) alleviated apoptosis induced by O_2_ in AEC II cells by Hoechst 33258 staining analysis

A: Representative pictures of apoptotic cells by Hoechst 33258 staining on day 3, 5 and 7; Control group: The cells were treated with normal medium alone; AS group: The cells were exposed to 50 μM AS; 80% O_2_-treated group: The cells were exposed to 80% O_2_; AS pretreatment group: The cells were treated with 50 μM AS followed by 80% O_2_. Scar bar=100 μm. (B) The ratio of apoptosis in AEC II cells. The red arrows indicate apoptotic cells. All data are represented as mean±SD, n=4 in each group. **P<0.01 vs control group, ##*P<*0.01 vs the cells 80% O_2_-treated group

**Figure 7:**
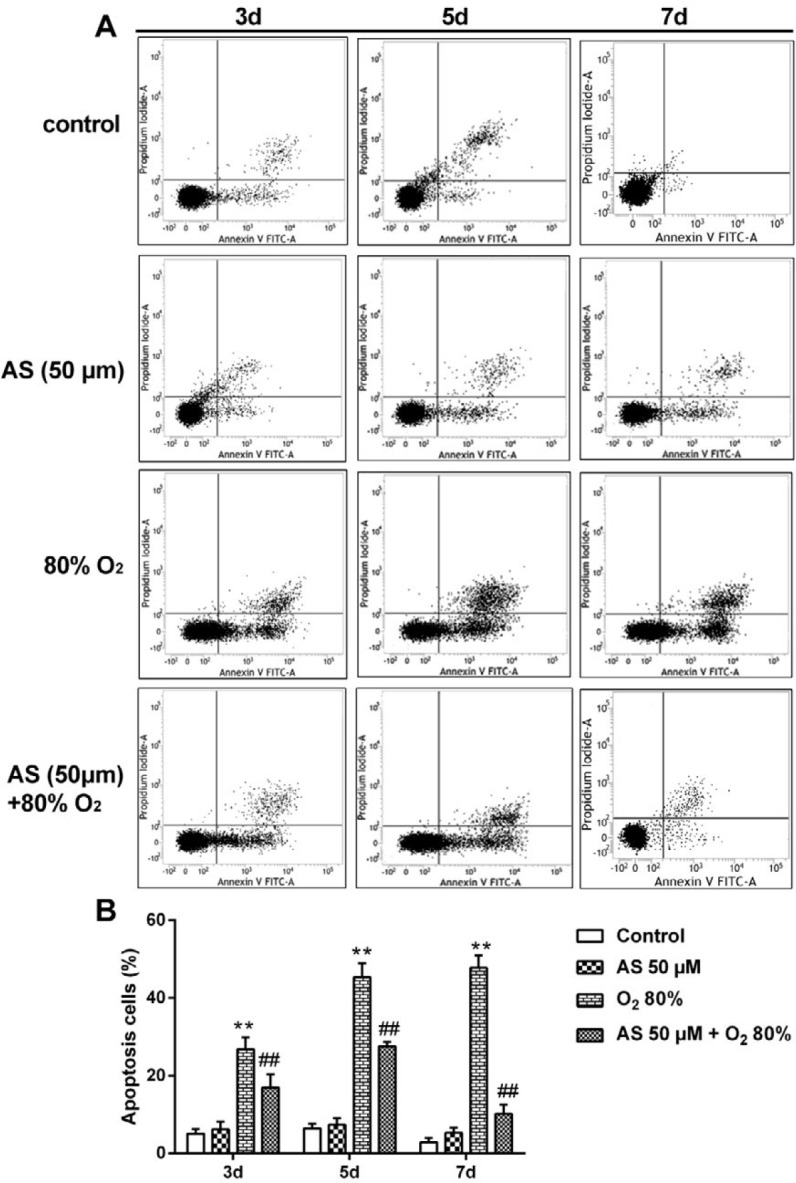
Asiaticoside (AS) alleviated apoptosis induced by O_2_ in AEC II cells with Annexin V-FITC/PI staining

(A) Representative pictures of apoptotic cells with Annexin V-FITC/PI staining at days 3, 5 and 7; Control group: The cells were treated with normal medium alone; AS group: The cells were exposed to 50 μM AS; 80% O_2_-treated group: The cells were exposed to 80% O_2_; AS pretreatment group: The cells were pretreatment with 50 μM AS followed by 80% O_2_. (B) Quantification of apoptotic ratio in each group. The results are represented as mean±SD, n=4 in each group. **P<0.01 vs control group, ##*P<*0.01 vs 80% O_2_-treated group

**Figure 8:**
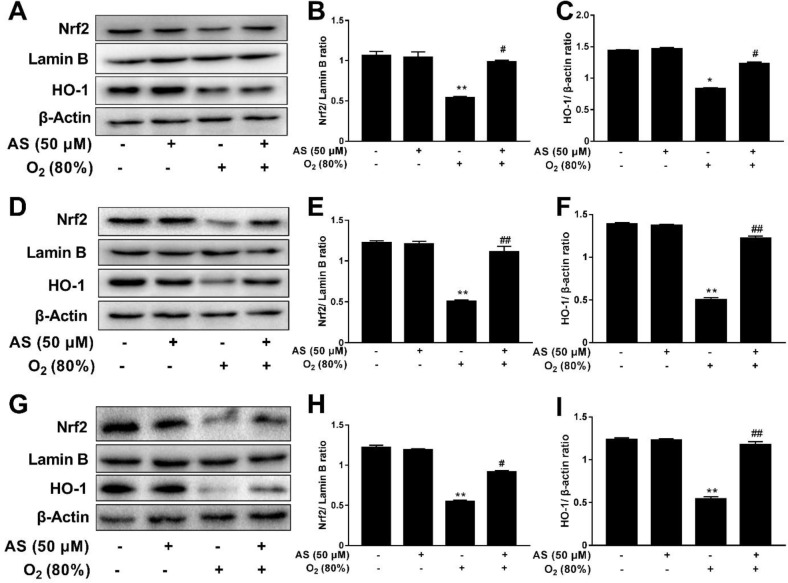
Asiaticoside up-regulated nuclear factor (erythroid-derived 2)-like 2 and heme oxygenase 1 expression in AEC II cells

(A) Western blot results of Nrf2 and HO-1 in AEC II cells exposed O_2_ (80%) with or without AS (50 μM) at day 3. (B-C) Quantification of western blot assay showing that O_2_ (80%) down-regulated Nrf2 and HO-1 expression, whereas AS treatment reversed Nrf2 and HO-1 level. (D) Western blot results of Nrf2 and HO-1 in AEC II cells exposed to O_2_ (80%) with or without AS (50 μM) at day 5. (E-F). Quantification of Western blot assay showing that O_2_ (80%) downregulated Nrf2 and HO-1 expression, whereas AS treatment reversed Nrf2 and HO-1 level. (G) Western blot results of Nrf2 and HO-1 in AEC II cells exposed O_2_ (80 %) with or without AS (50 μM) at day 7. (H-I). Quantification of Western blot assay showing that AS up-regulated Nrf2 and HO-1 expression after O_2_ (80%) exposure. The data are represented as mean±SD, n=4 in each group. *P<0.05 and **P<0.01 vs control group, #*P<*0.05 and ##*P<*0.01 vs O_2_ group

## Conclusion

Our studies illustrated that lung injury induced by hyperoxia contributed to development of BPD in premature rats. Hyperoxia could simulate specific manifestations inducing tissues injury in mature lung tissue, increasing apoptosis in lung tissue *in vivo* and AEC II cells *in vitro* and up-regulating MDA, MPO, TNF-α, IL-6, and IL-1β levels, but it decreased TAOC in premature rats. However, treatment with AS protected the lung from injury and attenuated apoptosis of lung tissues or AEC II cells by decreasing TNF-α, IL-6 , IL-1β, MDA, and MPO, but it up-regulated the expression of TAOC in the blood of premature rats. Moreover, the protective effect of AS was mediated by Nrf2/HO-1 signaling pathway. AS may have the potential to be a novel agent for the treatment of BPD clinically. 
